# HIV treatment default in sub-Saharan Africa: issues and possible solutions

**DOI:** 10.3389/fmed.2025.1688749

**Published:** 2025-10-15

**Authors:** Sandro Vento, Mbi Mbi, Massimiliano Lanzafame

**Affiliations:** ^1^Department of Clinical Disciplines, Manash Kozybayev North Kazakhstan University, Petropavlovsk, Kazakhstan; ^2^Faculty of Medicine, University of Puthisastra, Phnom Penh, Cambodia; ^3^Nyangabgwe Referral Hospital, Francistown, Botswana; ^4^Infectious Diseases Unit, Santa Chiara Hospital, Trento, Italy; ^5^Centre for Medical Sciences, University of Trento, Trento, Italy

**Keywords:** HIV, antiretrovirals, AIDS, treatment default, sub-Saharan Africa, traditional healers, stigma, patient-centered care

## 1 Introduction

The Minister of Health of South Africa stated on 15 May 2025 that 520,700 people living with HIV (PLHIV) had been initiated on antiretrovirals (ARVs) in the country as part of a campaign to start 1.1 million PLHIV on ARVs and achieve 95–95–95 targets by 31 December 2025 (1). However difficult it may be to initiate antiretroviral treatment in naïve individuals, keeping PLHIV on ARVs can be even harder. The KwaZulu-Natal Health Department in South Africa announced on 26 May 2025 that over 100,000 PLHIV in the province, including teenagers born with the virus, had defaulted on treatment (2). The persisting issue of treatment default is not limited to South Africa; it is particularly relevant in rural areas (3–5) and must be tackled.

## 2 Consequences of defaulting on treatment

Starting or continuing antiretrovirals is obviously not legally enforced, and it is therefore a personal decision based on correct information about the pros and cons of the treatment. For the individual PLHIV, the most serious possible consequence of ARV default is the progression to advanced HIV disease (AHD). In an analysis of data from population-based HIV impact assessment household surveys conducted between 2016 and 2021 among 28,040 PLHIV across 13 sub-Saharan African countries (Botswana, Cameroon, Côte d'Ivoire, Eswatini, Ethiopia, Lesotho, Malawi, Mozambique, Namibia, Tanzania, Uganda, Zambia, and Zimbabwe) to establish the proportion of adults with HIV who had AHD (i.e., CD4 cell count < 200/mm3), 7.6% (*n* = 1,871) of PLHIV were on ARVs but not virally suppressed (6). AHD was more common in male patients than the female ones (13.2% vs. 8.0%) (6). The highest proportion of people with AHD were those on ARVs but not virally suppressed [29.5% (95% CI: 26.6–32.6)]. The authors outlined that people with AHD who were on treatment with unsuppressed viral load might have recently initiated ARVs or re-initiated treatment after a period of default, which is quite common and associated with progression to AHD, as shown in South Africa (7).

Another important consequence of defaulting is the non-disclosure of prior treatment when people present to clinical facilities to restart ARVs. For instance, in South Africa, at least 45% of people starting ARVs have previous treatment experience, but only one-third of re-initiators voluntarily disclose this (8); this has obvious implications for drug resistance.

## 3 Proposed measures and issues to be overcome to curb the number of defaulting PLHIV

Several measures have been proposed: regular adherence counseling (9), facility adherence clubs, community adherence clubs, community ARV treatment groups, spaced fast lane appointments, community pick up points (10), food assistance, remote adherence monitoring, and SMS reminder systems. However, none of the above interventions can be easily and uniformly applied and maintained over time in any country. In our opinion, there are four main issues to be decisively faced: stigma, proper patient-centered care, religious beliefs, and the role of traditional healers.

## 4 Stigma

Stigma continues to be widespread in the communities and sometimes extends even to clinical settings. Many largely qualitative studies have shown that stigma contributes to poor retention in antiretroviral therapy in various sub-Saharan African countries (11–16), and a review article published in 2021 identified 14 articles showing substantial evidence of the negative effect of stigma on treatment adherence and viral suppression in different countries worldwide (17). In contrast, a secondary analysis of an HIV population in care in a high-prevalence rural community in South Africa found only a limited association between anticipated stigma or perceived level of community concerns about the infection and default from care (18). Stigma against people living with HIV among healthcare workers is not uncommon around the world, and sub-Saharan Africa is no exception (19, 20); importantly, disrespect in healthcare is absent from provider narratives, whereas patient interviews are filled with such reports (19). PLHIV must be treated with dignity and understanding, with an empathetic and non-judgmental approach and strong reassurance about strict confidentiality. To do this effectively, it is undoubtedly very important to move from words to actions and implement patient-centered care.

## 5 Patient-centered care

At a public-sector clinic in Johannesburg, South Africa, a few years ago, people failing second-line antiretroviral therapy were given the privilege to see the same clinician experienced in treatment failure during subsequent visits to maintain continuity of care and build a patient–provider bond (9). This continuity did not happen in the general clinic (9). It is essential that the same doctor and/or nurse follow the same person until either the former or the latter move to another clinic; this favors the development of an active partnership between the doctor and/or nurse and the individual PLHIV which allows the PLHIV to feel heard and understood, to be confident in explaining their emotional, psychological, and social issues and to become active in the decisions to be made. Over time, the doctor and/or the nurse will know well the person's lifestyle, their work, and their family responsibilities. Importantly, familiarity with their physicians allows people living with HIV to openly let them know whether they adhere to treatment, and how much so. The value of this aspect of care has been demonstrated in previous manuscripts from some of us (21, 22). Consultation time must be adequate, implying that all clinics are sufficiently staffed with doctors and nurses and that PLHIV are able to reach or contact them when necessary. Finally, if a person does not want to be followed at a clinic in their community because of the issue of stigma, opportunities to be followed at another clinic should be offered. Providing public health services in a respectful and friendly environment does not make services less effective; actually, the opposite is true (23).

Unfortunately, it is extremely difficult to implement patient-centered care in the public sector in sub-Saharan African countries. A main challenge is the high patient-to-health worker ratio, which obviously hugely restricts the time clinicians can dedicate to meaningful interactions with patients and to involving them in decision-making processes. Low staff motivation and inadequate supervision (24), scarce training in communication, poor accountability in the provision of care, and limited infrastructure to allow patient confidentiality and privacy protection (25, 26) are other serious challenges.

Satisfactory remuneration and improved working conditions are needed for doctors and nurses to provide adequate services. Salaries for public health services are often low, making it difficult to attract and retain healthcare workers, and the private sector and non-governmental organizations offer better pay and benefits, often attracting the best doctors and nurses from public health services. In addition to the high patient/staff ratio, a periodic lack of essential medical supplies often impedes effective treatment and care.

## 6 Religious beliefs

Spirituality is the path that individuals take to connect with their faith. In a systematic review evaluating the association between religion, spirituality and clinical outcomes in PLHIV, conducted for all English language articles published between 1980 and 2016, 67% of the studies reported a positive association between religion or spirituality and a clinical HIV outcome, 13% did not find such an association, and 13% revealed a negative association, possibly due to spirituality-induced distress (27). Religious leaders are highly regarded and viewed as trustworthy sources of information and guidance in sub-Saharan Africa. Therefore, their role and beliefs should not be underestimated. The relationship between religious leaders and HIV care in sub-Saharan Africa is particularly complex; some make claims that prayers can eliminate HIV, while others consider HIV/AIDS as God's punishment for sins such as being sexually promiscuous, and the vast majority are against the use of condoms as a prevention strategy, as they view condom use as a promotion of sexual immorality. However, various faith-linked organizations (e.g., Islamic Relief, Tear Fund, Caritas Internationalis, World Conference of Religion for Peace, and International Network of Religious Leaders living with HIV) help the WHO and UNAIDS in strengthening adherence to antiretrovirals and supporting PLHIV (28).

Christianity is one of the major religions in Africa, and Pentecostal Christians are the fastest-growing and the largest-growing movement in many regions (29). Quite a few Pentecostal pastors continue to be skeptical about the effectiveness of ARVs, as they only believe in spiritual healing and rather promote seeking divine intervention among their congregants to solve HIV-related issues (30). However, other pastors encouraged PLHIV on ARVs to continue treatment and access psychosocial support from other church members (28). In a cross-sectional study of 1,385 men who had sex with men registered in five key population clinics in five districts in South Africa in 2023 and 2024, Christian faith was a predictor of unsuppressed viral load in respect of no religion (31). In Zimbabwe, where a strong increase in faith healing organizations has been observed in recent years, many people with chronic conditions have joined them as their leaders have claimed to possess spiritual powers to heal illnesses; consequently, a number of PLHIV opted for spiritual healing over medical treatment and defaulted on antiretrovirals (32). In rural areas of the Democratic Republic of Congo, traditional churches urge PLHIV to start and continue treatment, whereas revival churches recommend using exorcism to cure the infection (33). In Kano, the main city of Northern Nigeria, which then had one of the highest levels of HIV prevalence in predominantly Muslim societies, research conducted 15 years ago found that controversies existed; some religious scholar-practitioners following the Islamic traditions of prophetic medicine thought that HIV could be completely cured if the individual had sufficient faith in the supernatural power of the Quran, others believed that the natural ingredients prescribed in Islamic texts could cure HIV, while many Muslim people on ARVs, and the Muslim healthcare workers treating them, were skeptic about whether a cure had yet to be revealed to people (34). However, in a study conducted in an HIV/AIDS clinic in the main Ugandan public hospital, a significantly positive relationship between ARV adherence and Pentecostal or Muslim religiosity (*r* = 0.618, *P* ≤ 0.01) was found (35). The above studies show that religious beliefs have an important role in ARV start and adherence in sub-Saharan Africa. Strong cooperation and mutual understanding must be actively pursued between religious leaders, doctors, and nurses to improve PLHIV's adherence to ARV treatment.

## 7 Role of traditional healers

In sub-Saharan Africa, traditional healers (THs) provide regular services to nearly 80% of the black African population, due to their accessibility and acceptability. In the case of HIV, the use of clinical treatment facilities may carry stigma in the community, in contrast to THs' consultation. Most PLHIV use traditional medicine together with Western medicine, and a considerable proportion of them use herbal medicine concomitantly with ARVs (36, 37). However, the majority of PLHIV rarely disclose their use of herbal medicines to doctors and nurses. Even though collaboration between THs, doctors, and nurses is essential, as trained and well-informed THs can successfully facilitate ARV adherence and retention in care for PLHIV (36), there are major challenges to cooperation: unstandardized herbal practices, the secrecy of THs, differing treatment philosophies, the absence of legal frameworks, and logistical barriers. Visiting a traditional healer is significantly related to incomplete ARV adherence in treatment-experienced adults in Tanzania, Uganda, and Zambia (38). In KwaZulu-Natal, South Africa, the use of traditional healthcare services by parents or primary caregivers of children was a barrier to ARV utilization (39), and in a study in Senegal, the strongest predictor of virologic failure was consulting a TH more than 6 months after ARV initiation [odd ratio (OR), 7.43; 95% CI, 1.22–45.24] (40). However, a TH support worker's intervention in rural Mozambique showed that traditional healers can provide community-based psychosocial support and education and directly observed therapy for PLHIV with poor adherence (41), and in a study conducted in rural Ethiopia, doctors and nurses recommended to establish collaboration through legal frameworks, registering genuine healers, protecting intellectual property, organizing regular forums for traditional healers' engagement, and fostering research partnerships (42). Issues within the use of both traditional healers and clinical facilities remain; for instance, PLHIV were willing to be tested for HIV by healers in rural South Africa trained in rapid, point-of-care HIV testing, but only 60% of those testing positive were enrolled in HIV treatment services (43).

## 8 Conclusion

Antiretroviral therapy defaulting remains a serious issue, which can lead to the development of drug resistance and to increased mortality, and is prominent among adolescents and youth (44). Despite many proposed and attempted solutions, defaulting remains too high; we believe that focusing on the four aspects that we have outlined could limit its occurrence.
